# Meta-analysis of virtual reality exposure therapy for social anxiety disorder

**DOI:** 10.1017/S0033291721001690

**Published:** 2023-04

**Authors:** Nexhmedin Morina, Isabel Kampmann, Paul Emmelkamp, Corrado Barbui, Thole H. Hoppen

**Affiliations:** 1 Institute of Psychology, University of Münster, Münster, Germany; 2 Department of Clinical Psychology, University of Amsterdam, Amsterdam, the Netherlands; 3 WHO Collaborating Centre for Research and Training in Mental Health and Service Evaluation, Department of Neuroscience, Biomedicine and Movement Sciences, Section of Psychiatry, University of Verona, Verona, Italy

Virtual reality exposure therapy (VRET) for social anxiety disorder (SAD) is a new technology-based form of exposure therapy that uses computer-generated virtual social environments as a means of systematically exposing patients to feared stimuli. An advantage of VRET relative to exposure *in vivo* is that virtual exposure takes place in a highly controllable environment. Recently, Horigome et al. (2020) published a meta-analysis on the efficacy of VRET for SAD and reported that 22 studies [11 randomized controlled trials (RCTs) and 11 non-randomized studies] fulfilled their inclusion criteria. We have previously systematically reviewed the literature on the efficacy of VRET for SAD (Carl et al., 2019; Emmelkamp, Meyerbröker, & Morina, 2020; Kampmann, Emmelkamp, & Morina, 2016b) and were surprised to read that a total 22 trials have investigated the efficacy of VRET for SAD. Therefore, we investigated with great interest potential reasons for the discrepancy in the number of RCTs in the review by Horigome et al. and our reviews.

Horigome and colleagues reported the following inclusion criteria for their meta-analyses: (1) a diagnosis of SAD, fear of public speaking, or public speaking anxiety; (2) VRET consisted of at least three sessions; and (3) study participants numbered a minimum of 10 patients. We critically evaluated the studies in the meta-analysis by Horigome et al. by applying their inclusion criteria and evaluating whether all of the treatments labeled as VRET did indeed consist of at least 50% of virtual reality components. As a result, we argue that the publication by Horigome and colleagues misleadingly implies that there is a larger body of literature on the efficacy of VRET for SAD than there actually is.

First, a clarification regarding the total number of included studies is needed. In both the abstract and the results section the authors reported that a total of 22 studies were included in their analyses. However, this number relates to the number of publications that the authors used for data extraction. A close look at Table 1 in Horigome et al. reveals that the authors counted the trial by Anderson et al. (2013) as well as the one by Safir, Wallach, and Bar-Zvi (2012) twice, respectively. Accordingly, they rather included a total of 20 studies described in 22 publications. Second, we argue that two of the included studies were erroneously labeled as VRET. First, the study by Lister et al. (2010^†^^1^) used virtual reality for assessment purposes only and their treatment did not comprise VRET. Second, the study by Yuen et al. (2019^1^) comprised of *in vivo* exposure in addition to VRET. More specifically, during sessions, participants were asked to engage in exposure to a real audience using video-conferencing. For homework, they received VRET while applying techniques of acceptance and commitment therapy and were encouraged to engage in at least three *in vivo* exposure exercises in-between sessions. Accordingly, <50% of treatment consisted of VRET and therefore any potential treatment efficacy might have resulted from other treatment elements than VRET (Morina, Ijntema, Meyerbröker, & Emmelkamp, 2015). Finally, six studies (Denizci Nazligul et al., 2019; Grillon et al., 2006; Harris et al., 2002; Kovar, 2018; North et al., 1998; Roy et al., 2003^1^) included <10 participants in VRET. Horigome et al. reported that ‘study participants numbered 10 or more people’, without specifying whether this number referred to the number of participants per condition or per trial. Research, however, strongly suggests that trials with small samples (i.e. low statistical power) reduce the likelihood that a statistically significant result reflects a true effect (Ioannidis, 2005). In fact, treatment effect estimates are often significantly larger in trails with small samples (Dechartres, Trinquart, Boutron, & Ravaud, 2013; Kjaergard, Villumsen, & Gluud, 2001; Turner, Bird, & Higgins, 2013). A minimum of 10 participants per condition was also applied in other related meta-analyses (e.g. Kampmann et al., 2016b; Morina et al., 2015). Consequently, we excluded the six trials with <10 patients per condition and argue that only 12 studies need to be included in the meta-analysis. In addition, Horigome et al. compared the efficacy of VRET to waitlist conditions and treatment as usual (TAU) combined. However, only one trial that used cognitive therapy as a comparison condition (Wallach, Safir, & Bar-Zvi, 2011) was defined as TAU and the remaining studies comprised waitlist conditions. Although it is questionable to categorize cognitive therapy as TAU, given their definition of cognitive behavioral therapy involving exposure *in vivo* as the gold standard SAD treatment, we also argue that it is clinically more useful to report on the efficacy of VRET as compared to waitlist only.

**Table 1.** Within and between group comparison results

Within-group ES	*k*^a^	*n*	*ES*	95% CI	*p*	*I*^2^	*Q*	*p*	PI
Baseline *v*.									
Post-treatment	12	228	1.20	0.91–1.50	<0.001	53.02	23.68	0.014	0.40–2.01
3 months FU	3	61	1.17	0.69–1.66	<0.001	5.42	3.03	0.220	0.47–1.87
6 months FU	2	38	1.19	0.70–1.68	<0.001	0.00	0.03	0.867	0.70–1.68
12 months FU	2	58	1.06	0.30–1.82	<0.001	73.31	3.75	0.053	−0.13 to 2.25
72 months FU	1	Number of trials too small							
Longest available FU^b^	5	110	1.06	0.71–1.41	<0.001	33.58	5.87	0.291	0.49–1.63
VRET *v*. waitlist									
At post-treatment	5	222	0.88	0.43–1.34	<0.001	61.34	10.16	0.038	0.03–1.79
VRET *v*. exposure *in vivo*									
at post	7	272	0.07	−0.32 to 0.17	0.562	10.65	6.57	0.362	−0.40 to 0.25
3 months FU	2	109	−0.64	−1.68 to 0.40	0.229	84.28	6.36	0.012	−2.34 to 1.07
6 months FU	1	Number of trials too small							
12 months FU	2	127	−0.02	−0.37 to 0.33	0.889	0.00	0.39	0.533	−0.37; 0.22
72 months FU	1	Number of trials too small							
Longest available FU^b^	4	206	−0.24	−0.83 to 0.35	0.426	76.70	11.21	0.011	−1.42 to 0.94

*k*, number of treatment arms; *n*, total number of participants in all relevant trials; PI, prediction interval.

*Note*: Effect sizes (ES) are reported as Hedges' *g*; the *I*^2^-statistic and *Q*-statistic are indicators of heterogeneity in percentages, with higher percentages indicating high heterogeneity.

a Number of treatment arms (i.e. *k*) is exactly the same as number of studies.

b Only the longest available follow-up data per trial was retained, except for Anderson et al. (2013) were the 12 months follow-up was retained rather than the exceptionally long 72 months follow-up.

In light of these limitations, we deemed it important to reanalyze the data on the efficacy of VRET for SAD. To this end, we included 12 studies in our meta-analysis, six of which were RCTs (Anderson et al., 2013; Bouchard et al., 2017; Kampmann et al., 2016a; Klinger et al., 2005; Safir et al., 2012; Wallach et al., 2011) and the remaining six were non-controlled studies (Anderson, Zimand, Schmertz, & Ferrer, 2007; Gebara, de Barros-Neto, Gertsenchtein, & Lotufo-Neto, 2016; Geraets et al., 2019; Kim et al., 2017; Robillard, Bouchard, Dumoulin, Guitard, & Klinger, 2010; Stupar-Rutenfrans, Ketelaars, & van Gisbergen, 2017).

To calculate uncontrolled effect sizes, the posttreatment or follow-up mean was subtracted from the pretreatment mean and for controlled effect sizes the control group mean was subtracted from the treatment group mean at posttreatment or follow-up, respectively, and divided by the pooled standard deviation. Subsequently, the outcome was multiplied by a sample size correction factor *J* = 1–(3/(4df − 1)) to obtain the effect size Hedges' *g*. Analyses were completed with the metafor package (v.1.9.8) in R 3.5 using random-effects models given the heterogeneity of the studies (R Core Team, 2015; Viechtbauer, 2010).

Detailed results of our analyses are presented in Table 1. Across all interventions, a large pre–post effect size was found. Effect sizes were further large when pre-assessment scores of social anxiety were compared to follow-up assessment scores at different time points. These findings indicate that VRET can significantly reduce symptoms of social anxiety. Note, however, that uncontrolled effect sizes do not account for the impact of time on symptoms, therefore controlled effect sizes need to be viewed as more reliable when it comes to assessing treatment efficacy. These analyses revealed that the pooled between-group effect size comparing VRET to waitlist at post-treatment was also large. Finally, the comparison of VRET to exposure *in vivo* yielded no significant differences between the two treatments, neither at post-assessment nor at follow-up.

When conducting our analyses on treatment efficacy at follow-up, we deviated from Horigome et al. to some extent. First, we did not conduct a meta-analysis with one trial only given the fact that a meta-analysis is per definition a technique that combines the results of multiple studies. In fact, in previous meta-analyses we have argued that a minimum of four trials is needed for a meaningful meta-analysis (Morina et al., 2015; Morina, Koerssen, & Pollet, 2016). Second and related to this, we decided to conduct an additional follow-up analysis with a larger amount of trials by including only the longest available follow-up data per trial [with the exception of Anderson et al. (2013) were the 12 months follow-up was retained rather than the exceptionally long 72 months follow-up]. As can be seen in Table 1, change from pretreatment to follow-up resulted in a large effect size across VRET treatments. Furthermore, there was no significant difference between VRET and exposure *in vivo* at follow-up.

Our within-group comparison findings are mostly in line with those reported by Horigome et al. Nonetheless, our findings show a stronger treatment efficacy of VRET from pre-treatment to post-treatment and to follow-up. With respect to between-group comparisons, our results vary from those reported by Horigome et al. to some greater extent than the within-group findings. First, the comparison of VRET to wait-list is smaller in our meta-analysis, albeit still in the range of a large effect. Second, our analyses of the efficacy of VRET relative to exposure *in vivo* produced insignificant results only, whereas Horigome et al. report significant results between VRET and exposure *in vivo* at follow-up that go in both directions. Yet, we need to interpret these results with caution as these are based on a limited number of RCTs.

## Footnotes

† The notes appear after the main text.

## Notes

1 Due to the word limit, we refer here to the reference list in Horigome at al. (2020).
